# Reconsidering treatment guidelines for acute myocardial infarction during the COVID-19 pandemic

**DOI:** 10.1186/s12872-022-02626-5

**Published:** 2022-04-26

**Authors:** Jing Gao, Peng-Ju Lu, Chang-Ping Li, Hui Wang, Ji-Xiang Wang, Nan Zhang, Xiao-Wei Li, Hai-Wang Zhao, Jing Dou, Miao-Na Bai, Yu-Tian Shi, Jia Zhao, Chun Zan, Yin Liu

**Affiliations:** 1 Chest Hospital, Tianjin University, No. 92 Weijin Road Nankai District, Tianjin, 300072 People’s Republic of China; 2 Thoracic Clinical College, Tianjin Medical University, No. 22 Qi xiang tai Road, Heping District, Tianjin, 300070 People’s Republic of China; 3 Cardiovascular Institute, Tianjin Chest Hospital, No. 261 Tai er zhuang Road, Jinnan District, Tianjin, 300222 People’s Republic of China; 4 Department of Cardiology, Tianjin Chest Hospital, No. 261 Tai er zhuang Road, Jinnan District, Tianjin, 300222 People’s Republic of China; 5 Tianjin Medical University, No.22 Qi xiang tai Road, Heping District, Tianjin, 300070 People’s Republic of China

**Keywords:** Coronavirus disease-2019, Acute myocardial infarction, First medical contact time, Major adverse cardiac event

## Abstract

**Background:**

COVID-19 affects healthcare resource allocation, which could lead to treatment delay and poor outcomes in patients with acute myocardial infarction (AMI). We assessed the impact of the COVID-19 pandemic on AMI outcomes.

**Methods:**

We compared outcomes of patients admitted for acute ST-elevation MI (STEMI) and non-STEMI (NSTEMI) during a non-COVID-19 pandemic period (January–February 2019; Group 1, n = 254) and a COVID-19 pandemic period (January–February 2020; Group 2, n = 124).

**Results:**

For STEMI patients, the median of first medical contact (FMC) time, door-to-balloon time, and total myocardial ischemia time were significantly longer in Group 2 patients (all *p* < 0.05). Primary percutaneous intervention was performed significantly more often in Group 1 patients than in Group 2 patients, whereas thrombolytic therapy was used significantly more often in Group 2 patients than in Group 1 patients (all *p* < 0.05). However, the rates of and all-cause 30-day mortality and major adverse cardiac event (MACE) were not significantly different in the two periods (all *p* > 0.05). For NSTEMI patients, Group 2 patients had a higher rate of conservative therapy, a lower rate of reperfusion therapy, and longer FMC times (all *p* < 0.05). All-cause 30-day mortality and MACE were only higher in NSTEMI patients during the COVID-19 pandemic period (*p* < 0.001).

**Conclusions:**

COVID-19 pandemic causes treatment delay in AMI patients and potentially leads to poor clinical outcome in NSTEMI patients. Thrombolytic therapy should be initiated without delay for STEMI when coronary intervention is not readily available; for NSTEMI patients, outcomes of invasive reperfusion were better than medical treatment.

## Highlights

- COVID-19 pandemic causes treatment delay for AMI and leads to poor clinical outcome.
- Thrombolytic therapy should be initiated without delay for STEMI when coronary intervention is not readily available.
- For NSTEMI patients, outcomes of invasive reperfusion were better than medical treatment.
- All hospitals must accommodate expeditious and appropriate care for AMI during COVID-19 pandemic.

## Background

On March 11, 2020, the World Health Organization announced that the new coronavirus disease 2019 (COVID-19) had become a global pandemic. How to balance the care of cardiovascular emergency patients and the control of COVID-19 has become a global challenge during the pandemic. For patients with acute myocardial infarction (AMI) who also have COVID-19, a safe and effective medical environment is required in parallel with effective reperfusion therapy. Based on the experience of AMI management during the COVID-19 pandemic, Peking Union Medical College Hospital has made medical recommendations for China [1]. These suggestions have been confirmed by the practice of many Chinese medical institutions [2, 3] and should be promoted globally for peer reference [4, 5]. These recommendations strictly abide by the COVID-19 prevention principles of the World Health Organization and the regulations of the national health authorities.

Accordingly, our hospital initiated response measures in January 2020 to combat the COVID-19 epidemic and revised the reperfusion treatment guidelines for AMI patients. However, at the same time, appropriate but important restrictions have been imposed on routine medical care to comply with the regulations on physical alienation in the public health guidelines and to help conserve or redirect limited resources. During this time, most invasive cardiovascular procedures and diagnostic tests have been postponed, and the North American Cardiovascular Society advocates the classification of patients and the management of patients on waiting lists [6]. Unfortunately, patients with untreated cardiovascular disease are at increased risk of adverse outcomes [7]. A recent report showed that COVID-19 may affect human behavior and the allocation of health care resources, leading to delays in the treatment of AMI patients [8]. Delays in treatment for patients with confirmed cardiovascular disease will be harmful. In addition, reducing access to diagnostic tests will result in a high burden of undiagnosable cardiovascular diseases, which will further delay treatment. However, there are no studies evaluating the impact of the revised guidelines on treatment outcomes for AMI patients, although several studies have noted reduced hospitalization [9], untreated mortality [10], incidence [7], systemic inflammation and hypercoagulability [11], disease manifestations and care [12] and delayed treatment [13] in patients with AMI. Therefore, we investigated the impact of the COVID-19 pandemic on AMI outcome in our patients (Tianjin, China).

Accordingly, our hospital launched emergency response measures in January 2020 to combat the COVID-19 outbreak, and it revised guidelines for reperfusion therapy in AMI patients. However, concurrently, appropriate but significant restrictions have been made on routine medical care to comply with public health guidance on physical distancing and to help preserve or redirect limited resources. During this time, most invasive cardiovascular procedures and diagnostic tests have been deferred, with North American cardiovascular societies advocating for intensified triage and management of patients on waiting lists [6]. Unfortunately, patients with untreated cardiovascular disease are at increased risk of adverse outcomes [7]. A recent report revealed that COVID-19 could affect human behavior and healthcare resource allocation which led to treatment delay in AMI patients [8]. Delays in the treatment of patients with confirmed cardiovascular disease will be detrimental. Moreover, reduced access to diagnostic testing will lead to a high burden of undiagnosed cardiovascular disease that will further delay treatment. As healthcare systems return to normal capacity, the risk to cardiovascular patients will warrant prioritizing them among the competing demands of a myriad of patients with other medical problems [6]. Therefore, we investigated the impact of the COVID-19 pandemic on AMI outcome in our patients (Tianjin, China).

## Materials and methods

### Patient and public involvement

The study protocol was approved by the Ethics Committee of our Hospital (No. 2018KY-010-01), and all study steps were conducted under the supervision of this committee. All the patients were provided with written and oral explanations on the study course and goals. Participants then gave written informed consent before enrollment. Moreover, this study was conducted in accordance with the Declaration of Helsinki. This cross-sectional, single-center study enrolled patients with AMI diagnosed according to the Fourth Universal Definition of Myocardial Infarction [14] and the onset of AMI less than 72 h in the cardiac/coronary care unit of our hospital. Exclusion criteria: (1) patients with chest pain who did not meet the diagnostic criteria of AMI and (2) patients with AMI who did not cooperate with treatment. Patients were divided into Group 1 (non-COVID-19 pandemic period, 254 cases), who were admitted 24 January 2019 to 28 February 2019 and Group 2 (COVID-19 pandemic period, 124 cases), who were admitted 24 January 2020 to 29 February 2020. Group 1 included 153 patients with ST-segment elevation myocardial infarctions (STEMI) and 101 patients with non-ST-segment elevation myocardial infarctions (NSTEMI). Group 2 included 81 STEMI and 43 NSTEMI patients. All data for follow-up were collected retrospectively from the emergency department electronic medical record system and the in-patient electronic medical record system, which included demographic characteristics, previous medical history, clinical examination and treatment, and medical outcomes.

### The treatment process during the COVID-19 pandemic

**Fig. 1:**
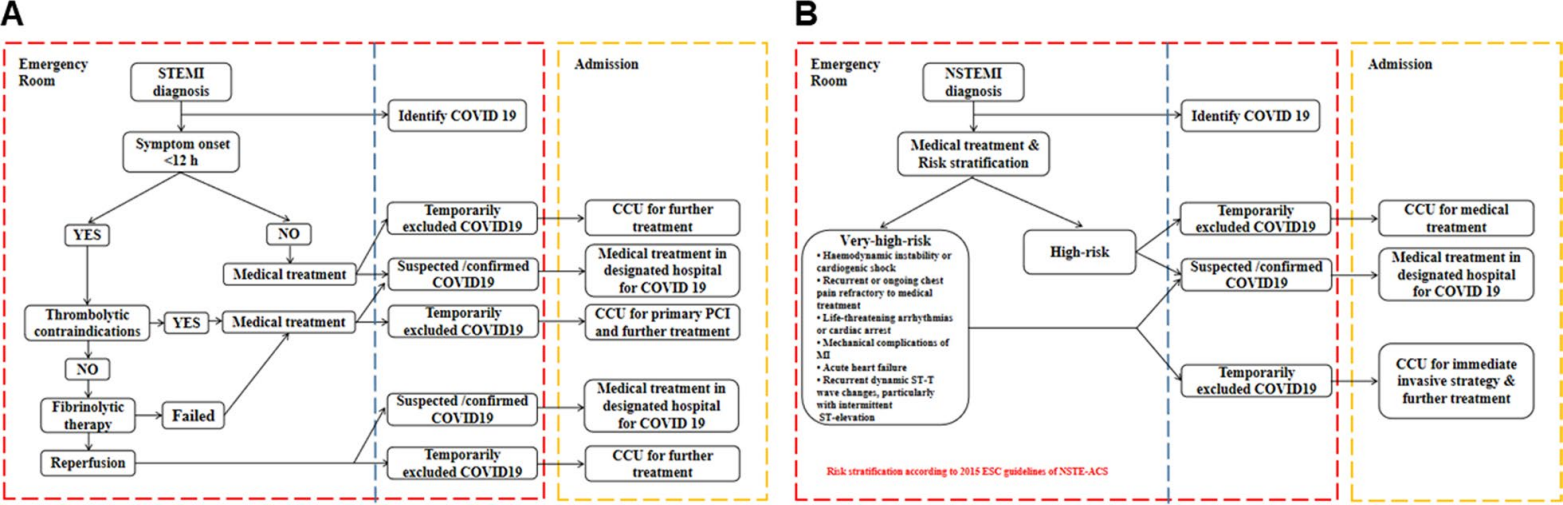
Management of suspected AMI patients during the COVID-19 pandemic. **a** STEMI patients. **b** NSTEMI patients

According to the COVID-19 trend and current best guidelines for AMI treatment [15, 16], our Hospital formulated a modified process (24 January 2020) for management of AMI during the COVID-19 pandemic. As shown in Fig. 1, patients with diagnosed STEMI or NSTEMI were simultaneously assessed for COVID-19 infection according to laboratory tests, chest radiograph, and chest computed tomography in the emergency department. Patients with STEMI diagnosed within 12 h were given the latest generation specific thrombolytic drugs (recombinant human urokinase for injection, puyouke [Tianshi biomedical, China]) for thrombolytic therapy. STEMI patients who had contraindications to thrombolysis or failure of thrombolysis, had COVID-19 infection excluded. Those who had perceived benefit over risk of percutaneous coronary intervention (PCI) received PCI in a designated catheter room that meets requirements; those with suspected or confirmed COVID-19 were treated conservatively with medications. Patients whose onset time of STEMI was more than 12 h and those with suspected or conformed COVID-19 received conservative treatment, while patients with COVID-19 infection excluded and a perceived benefit of PCI over the risk received PCI. NSTEMI patients first received conservative treatment, whereas patients with COVID-19 excluded and a high risk of disease according to risk stratification received PCI after the assessment for PCI risk and benefit. None of the patients had findings indicative of active COVID-19 infection.

### COVID-19 screening

Prior to receiving any cardiovascular procedure or test, patients received routine screening to help ensure the safety of health care workers. The testing included nasopharyngeal swab nucleic acid test according to the manufacturer’s instructions.

### Statistical analysis

The Kolmogorov–Smirnov test was used to verify the normality of all continuous variables. Continuous variables were reported as mean ± standard deviation (SD) or median and interquartile range (IQR), as appropriate. Categorical values were presented as absolute values or percentages. Comparison of continuous variables was performed with one-way ANOVA, the Mann–Whitney *U* test, or the independent samples Kruskal–Wallis test, whereas the Chi-square or Fisher's exact tests were used for comparison of categorical values, depending on the distribution of data. The Kaplan–Meier method was used to compare the MACE-free survival time and all-cause death-free survival time for patients with non-COVID-19 and COVID-19, and curves were compared using the log-rank test. All statistical assessments were two-tailed, were considered significant at a *p*-value < 0.05, and were performed with IBM SPSS statistical software version 25 for Windows (IBM Corp., New York, USA).

## Results

### Patient characteristics

Patients’ baseline characteristics are presented in Table 1. One hundred fifty-three STEMI and 101 NSTEMI patients were included in group 1 (non-COVID-19 pandemic period), and 81 STEMI and 43 NSTEMI patients were included in group 2 (COVID-19 pandemic period), with median age of 64 years in both groups. The baseline characteristics of age, gender, diabetes mellitus, hypertension, prior coronary artery disease, prior MI, prior PCI, prior coronary artery bypass graft (CABG), smoking status, alcohol use, blood pressure, left ventricular ejection fraction, Killip class, and GRACE score, were similar in the two groups (all *p* > 0.05), although the heart rate was significantly higher in group 2 patients than in group 1 patients (*p* < 0.015).

**Table 1:** Baseline characteristics of patients with acute myocardial infarction in non-COVID-19 and COVID-19 pandemic periods

	AMI			STEMI			NSTEMI		
	Group 1 (n = 254)	Group 2 (n = 124)	*p-*value	Group 1 (n = 153)	Group 2 (n = 81)	*p-*value	Group 1 (n = 101)	Group 2(n = 43)	*p-*value
Age, years, mean ± SD/median(IQR)	64 (57.0,72.0)	64 (55.3,70.0)	0.306	63.1 ± 12.6	62.7 ± 11.6	0.809	68.0 (59.0,72.0)	66.0 (56.0,71.0)	0.211
Gender/male, n (%)	190 (74.8)	95 (76.6)	0.701	114 (74.5)	62 (76.5)	0.732	76 (75.3)	33 (76.7)	0.848
Diabetes mellitus, n (%)	86 (35.5)	36 (34.3)	0.823	48 (32.0)	26 (34.7)	0.688	38 (41.3)	10 (33.3)	0.438
Hypertension, n (%)	157 (64.9)	62 (59.0)	0.301	98 (65.3)	45 (60.0)	0.433	59 (64.1)	17 (56.7)	0.464
Prior MI, n (%)	14 (5.8)	11 (10.5)	0.121	3 (2.0)	4 (5.3)	0.342	11 (12.0)	7 (23.3)	0.219
Prior CAD, n (%)	11 (4.5)	5 (4.8)	0.930	3 (2.0)	2 (2.7)	1.000	8 (8.7)	3 (10.0)	1.000
Prior PCI, n (%)	18 (92.6)	11 (10.5)	0.348	9 (6.0)	6 (8.0)	0.571	9 (9.8)	5 (16.7)	0.486
Prior CABG, n (%)	7 (2.9)	3 (2.9)	0.986	4 (2.7)	1 (1.3)	0.873	3 (3.3)	2 (6.7)	0.774
Smoker, n (%)	154 (71.6)	61 (28.4)	0.329	96 (64.0)	40 (53.3)	0.123	58 (63.0)	21 (70.0)	0.489
Alcohol use, n (%)	75 (31.0)	37 (35.2)	0.437	47 (31.3)	26 ( 34.7)	0.615	28 (30.4)	11 (36.7)	0.525
Systolic pressure (mmHg), mean ± SD/median (IQR)	139 (126.0,159.5)	145 (123.0,160.0)	0.476	141.1 ± 23.2	145.3 ± 30.3	0.288	139.0 (125.5,160.0)	142.0 (125.0,154.0)	0.441
Diastolic pressure (mm Hg), mean ± SD/median (IQR)	82 (72.0,95.0)	87 (73.8,98.0)	0.101	85.0 ± 16.9	88.3 ± 18.2	0.168	82.0 ± 14.4	84.3 ± 17.2	0.416
Heart rate (beats per min), median (IQR)	76 (66.0,90.0)	80.5 (71.0,93.0)	0.017^*^	75.0 (64.0,89.0)	80.0 (69.0,88.0)	0.047	78.0 (70.0,91.0)	82.0 (76.0,100.0)	0.015
cTNI (ng/mL),median (IQR)	1.20 (0.63,2.46)	1.23 (0.65,3.08)	0.618	1.14 (0.60,2.16)	1.23 (0.69,3.62)	0.188	1.22 (0.65,2.76)	1.10 (0.36,2.13)	0.377
LVEF, median (IQR)	50 (42.3,56.0)	50 (41.0,56.0)	0.647	48.0 (42.0,55.0)	50.0 (42.0,55.0)	0.368	55.0 (45.0,57.0)	52.0 (42.0,56.0)	0.193
Arrhythmia, n (%)	19 (7.4)	11 (8.9)	0.639	11 (7.2)	8 (9.9)	0.474	8 (7.9)	3 (7.0)	0.845
Atrial fibrillation	8 (3.1)	4 (3.2)		5 (3.3)	3 (3.7)		3 (3.0)	1 (2.3)	
Ventricular tachycardia	4 (1.6)	3 (2.4)		2 (1.3)	2 (2.5)		2 (2.0)	1 (2.3)	
Ventricular fibrillation	3 (1.2)	2 (1.6)		2 (1.3)	2 (2.5)		1 (1.0)	0 (0)	
Atrioventricular block III	4 (1.6)	2 (1.6)		2 (1.3)	1 (1.2)		2 (2.0)	1 (2.3)	
Killip class, n (%)									0.084
1	218 (95.6)	109 (94.0)	0.482	136 (97.1)	74 (98.7)	0.611	82 (93.2)	35 (85.4)	
2	8 (3.5)	3 (2.6)			2 (1.4)	0 (0.0)		6 (6.8)	3 (7.3)
3	1 (0.4)	3 (2.6)			1 (0.7)	1 (1.3)		0 (0.0)	2 (4.9)
4	1 (0.4)	1 (0.9)			1 (0.7)	0 (0.0)		0 (0.0)	1 (2.4)
Very-high risk^#^, n (%)							19 (18.8)	13 (30.2)	0.131

*COVID-19* coronavirus disease 2019, *AED* accident and emergency department, *CABG* coronary artery bypass graft, *ECG* electrocardiogram, *MI* myocardial infarction, *NSTEMI* non-ST elevation myocardial infarction, *PCI* percutaneous coronary intervention, *STEMI* ST elevation myocardial infarction, *LVEF* left ventricular ejection fraction, *cTNT* cardiac troponin T, *IQR* interquartile range

^#^Risk stratification according to 2015 ESC guidelines of NSEMI *P < 0.05

### Clinical treatment

Clinical treatment of STEMI patients during the non-COVID-19 pandemic period (Group 1) and the COVID-19 pandemic period (Group 2) is presented in Table 2. There were trends towards more frequent use of reperfusion therapy in Group 1 than in Group 2 patients and more frequent use of conservative therapy in Group 2 patients than in Groups 1 patients, but these trends did not reach statistical significance. However, primary PCI was performed significantly more often in Group 1 patients than in Group 2 patients, whereas thrombolytic therapy and selective PCI was used significantly more often in Group 2 patients than in Group 1 patients (all *p* < 0.05). The median time to first medical contact was about twice as long in Group 2 patients as in Group 1 patients, and the median door-to-balloon (DTB) time also was longer in the Group 2 patients. Total myocardial ischemia time was about twice longer in Group 2 patients than in Group 1 patients (all *p* < 0.05).

**Table 2:** Comparison of clinical treatment between STEMI patients during non-COVID-19 and COVID-19 pandemic periods

	STEMI		*p-*value
	Group 1 (n = 153)	Group 2 (n = 81)	
**Treatment place, n (%)**			
Accident and emergency department	3 (2.0)	6 (7.4)	0.088
In-hospital	150 (98.0)	75 (92.6)	
**Reperfusion therapy, n (%)**	**115 (75.2)**	**55 (67.9)**	0.236
Primary PCI	100 (65.4)	20 (24.7)	< 0.001*
Thrombolytic therapy	6 (3.9)	21 (25.9)	< 0.001*
Selective PCI	9 (5.9)	14 (17.3)	0.005*
CABG	0 (0.0)	0 (0.0)	-
**Conservative therapy, n (%)**	**38 (24.8)**	**26 (32.1)**	0.236
**First medical contact time (min), median (IQR)**	111.0(55.0,282.0)	223.5 (118.8,567.3)	< 0.001*
**DTB time (min), median (IQR)**	55.0(48.0,66.0)	67.5(50.3,116.3)	0.021*
**Total myocardial ischemia time (min), median (IQR)**	189.0(118.0,338.0)	383.5(198.0,654.0)	0.018*

*STEMI* ST elevation myocardial infarction, *COVID-19* coronavirus disease 2019, *DTB* door-to-balloon, *PCI* percutaneous coronary intervention, *CABG* coronary artery bypass graft, *IQR* interquartile range

*p < 0.05

Comparison of clinical treatment during the non-COVID-19 pandemic period (Group 1) and the COVID-19 pandemic period (Group 2) NSTEMI patients is presented in Table 3. In contrast with treatment of patients who had STEMI, the differences in rates of reperfusion therapy and conservative treatment were significantly different between Group 1 patients and Group 2 (*p* < 0.05). As with STEMI patients, though, median FMC times were significantly longer in Group 2 NSTEMI patients than in Group 1 NSTEMI patients (*p* < 0.05).

**Table 3:** Comparison of clinical treatment between NSTEMI patients during non-COVID-19 and COVID-19 pandemic periods

	NSTEMI		*p-*value
	Group 1 (n = 101)	Group 2 (n = 43)	
**Treatment place, n (%)**			0.001*
Accident and emergency department	9 (8.9)	13 (30.2)	
In-hospital	92 (91.1)	30 (69.8)	
**Reperfusion therapy, n (%)**	71 (70.3)	16 (37.2)	< 0.001*
PCI within 24 h in-hospital	18 (17.8)	9 (20.9)	0.662
PCI after 24 h in-hospital	44 (43.6)	7 (16.3)	0.002*
CABG	9 (8.9)	0 (0.0)	0.100
**Conservative therapy, n (%)**	30 (29.7)	27 (62.8)	< 0.001*
**FMC time (min), median (IQR)**	236.0 (106.0,675.0)	412.0 (181.0,839.0)	0.036*

*NSTEMI* non-ST elevation myocardial infarction, *COVID-19* coronavirus disease 2019, *FMC* first medical contact, *PCI* percutaneous coronary intervention, *CABG* coronary artery bypass graft, *IQR* interquartile range.

*p < 0.05

### Patient outcomes in the non-COVID-19 pandemic period and the COVID-19 pandemic period

Thirty-day outcomes of STEMI and NSTEMI patients in the non-COVID-19 pandemic period and the COVID-19 pandemic period are presented in Table 4. For STEMI patients, slightly but not statistically significant higher rates of all-cause mortality were recorded in the COVID-19 pandemic period (Group 2); rates of major adverse cardiac event (MACE), all-cause death with MACE, recurrent AMI and revascularization also were not significantly different in the two periods (all *p* > 0.05). In contrast with STEMI patients, NSTEMI patients in the COVID-19 pandemic period (Group 2) had significantly higher rates of all-cause mortality, MACE, and MACE-associated all-cause mortality than did patients in the non-COVID-19 pandemic period (all *p* < 0.05).

**Table 4:** Outcomes of STEMI and NSTEMI patients in 30-day follow-up

	STEMI			NSTEMI		
	Group 1 (n = 153)	Group 2 (n = 81)	*p-*value	Group 1 (n = 101)	Group 2 (n = 43)	*p-*value
**All-cause mortality, n (%)**	5 (3.3)	6 (7.4)	0.363	3 (3.0)	8 (18.6)	< 0.003*
**MACE, n (%)**	9 (5.9%)	8 (9.9%)	0.534	5 (5.0%)	8 (18.6%)	0.020*
All-cause death	5 (3.3)	6 (7.4)	0.363	3 (3.0)	8 (18.6)	< 0.003*
Recurrent AMI	0 (0.0)	0 (0.0)	–	0 (0.0)	0 (0.0)	–
Heart failure	1 (0.7)	2 (2.6)	0.580	0 (0.0)	0 (0.0)	–
Revascularization	3 (2.1)	0 (0.0)	0.502	2 (2.2)	0 (0.0)	0.910
Stroke	0 (0.0)	0 (0.0)	–	0 (0.0)	0 (0.0)	–

*STEMI* ST elevation myocardial infarction, *NSTEMI* non-ST elevation myocardial infarction, *MACE* major adverse cardiac event, *MI* myocardial infarction.

*p < 0.05

**Fig. 2:**
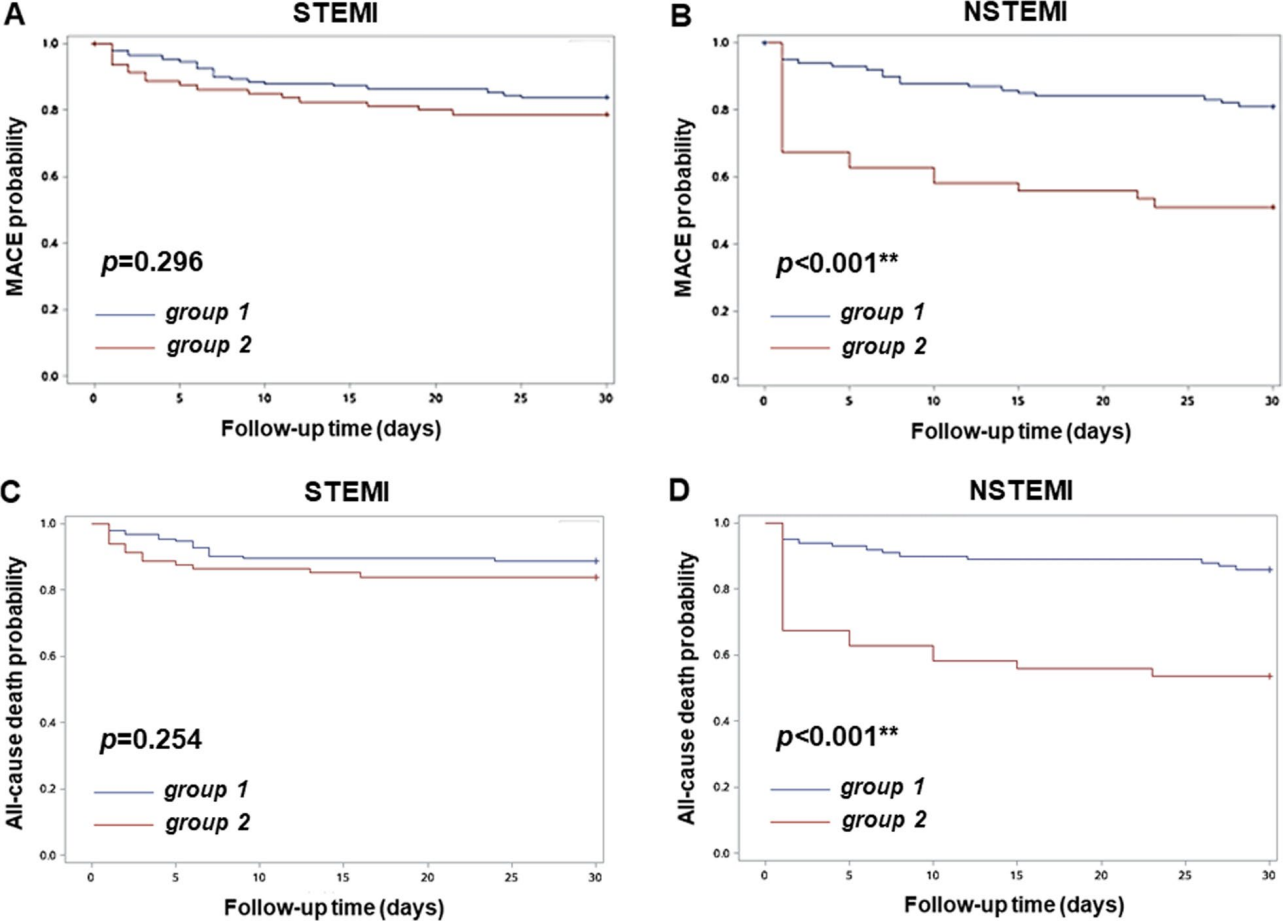
Kaplan–Meier estimates of the risk of MACE and all-cause mortality during the non-COVID-19 and COVID-19 pandemic periods. Comparison of 30-day major adverse cardiovascular event-free survival rate between **a** STEMI and **b** NSTEMI patients in the non-COVID-19 and COVID-19 pandemic periods. Comparison of 30-day all-cause death-free survival rate between **c** STEMI and **d** NSTEMI patients in the non-COVID-19 and COVID-19 pandemic periods

In Kaplan–Meier plot analysis (Fig. 2), STEMI patients had similar probability of having MACE and all-cause death during the non-COVID-19 pandemic period (Group 1 patients) and the COVID-19 pandemic periods (Group 2 patients). In contrast, NSTEMI patients had significantly higher probably of having MACE and all-cause death in the COVID-19 pandemic period than in the non- COVID-19 pandemic period (all *p* < 0.05).

## Discussion

This study analyzed the management of patients with acute coronary artery disease during a two-month period of the COVID-19 pandemic compared with management during a similar two-month period a year earlier. Substantial differences—some with important effects on patient outcomes—were identified: For NSTEMI patients, reperfusion therapy was used significantly less often in the pandemic period, whereas conservative treatment was used significantly more often, and first medical contact times were longer. Correspondingly, NSTEMI patients had significantly higher probability of MACE, MACE-associated death, and all-cause death in the COVID-19 pandemic period than in the non-COVID-19 pandemic period. These findings imply that delays in initiating cardiac care imposed by COVID-19 resulted in poorer outcomes for NSTEMI. In contrast with NSTEMI patients, rates of reperfusion therapy and conservative therapy for STEMI patients were not significantly different between the non-pandemic and pandemic periods; also, although FMC and total myocardial ischemia times for STEMI patients were much longer in the pandemic period than in the non-pandemic period, the probabilities of MACE, MACE-associated death, and all-cause death were not significantly different in the two periods. Ours is the only study we know of that related conservative therapy, FMC time, and prognosis in NSTEMI patients during the COVID-19 pandemic and non-COVID-19 periods. We also know of no study that has correlated the high risk of all-cause mortality and MACE with the increased FMC time and conservative therapy of NSTEMI patients during these two periods.

The COVID-19 pandemic has led to marked global morbidity and mortality [17–19]. Guidelines for the treatment of AMI had to be modified during the COVID-19 pandemic period. A worrying reduction in admissions for AMI was observed across many countries, with a parallel increase in symptom-to-balloon time, total ischemic times, case fatality, and complication rates [7, 9–13, 20]. Robust evidence shows that dual antiplatelet therapy (DAPT), consisting of aspirin and an oral P2Y12 inhibitor, mitigates the incidence of ischemic events [21, 22]. A meta-analysis study supports a paradigm shift in antithrombotic management, and it questions the central role of DAPT beyond one to three months after PCI [23]; these observations indicate that the appropriate use of antithrombotic therapy is vital to balance treatment benefit vs risks and improve outcomes. According to the North American cardiovascular societies [6, 24] and our hospital guidelines, invasive cardiovascular procedures and diagnostic tests for AMI patients during the COVID-19 pandemic are deferred (Fig. 1). Simultaneously, thrombolytic therapy is the first choice for the treatment of STEMI patients during the COVID-19 pandemic, while selective PCI is the secondary choice when these patients have thrombolytic contraindications or are age > 75 years (Fig. 1A). Hence, our data showed that therapy and selective PCI were used significantly more often in STEMI patients during the COVID-19 pandemic than in STEMI patients during the non-COVID-19 pandemic. However, fewer percutaneous coronary interventions have increased the risk of adverse outcomes during the COVID-19 pandemic [7, 9, 25]. Similarly, reduced access to diagnostic testing likely will lead to a high burden of undiagnosed cardiovascular disease that will further delay time to treatment [6, 12, 13, 20]. In agreement with this opinion, our STEMI patients had significantly fewer primary PCI and significant longer FMC time, door-to-balloon (DTB) time, and total myocardial ischemia time during the COVID-19 pandemic period.

Prompt diagnosis and treatment can reduce mortality, improve prognosis, and reduce the duration of hospital stay in patients with STEMI [26]. Reperfusion therapy should be started as soon as possible—at the latest within 90 min from FMC [27]. In the present study, FMC time about doubled in STEMI patients during the COVID-19 pandemic period compared with that in the non-COVID-19 pandemic period. Despite this change, the frequency of conservative therapy, all-cause mortality, and MACE in STEMI patients during COVID-19 pandemic period did not significantly increase, whereas NSTEMI patients had increase FMC time and frequency of conservative therapy use during the COVID-19 pandemic period. Subsequent results indicated that all-cause mortality and MACE were also increased in NSTEMI patients during the COVID-19 pandemic period. Thus, we speculate that the increased FMC time-related prognosis in NSTEMI was due to the increase in the use of conservative therapy during the COVID-19 pandemic period. A meta-analysis, covering seven trials with up-to-date adjunctive medication, showed a significant reduction in risk for all-cause mortality and myocardial infarction in NSTEMI patients for an early invasive vs. conservative approach at two years but no increase in death rate or myocardial infarction at one month [28]. Another meta-analysis, of eight randomized clinical trials, also found a significantly lower incidence of death, myocardial infarction, and rehospitalization at one year for NSTEMI patients who received invasive strategy [29]. These studies support our view that poor outcomes in our patients were due mainly to the increased use of conservative therapy during the COVID-19 pandemic period. Consequently, our results suggest that a routine invasive strategy for NSTEMI patients during the COVID-19 pandemic period was appropriate, while highlighting the importance of risk stratification in the decision-making. However, our study didn't include active SARS-CoV-2 infected patients, and it was not about the management of acute myocardial infarction in COVID-19 patients.

Timely implementation of reperfusion therapy is key in the management of STEMI, since the greatest benefit gained from reperfusion therapy occurs within the first two–three hours of symptom onset [26, 30]. Short total ischemic time—between symptom onset and initiation of reperfusion therapy (either starting thrombolysis or performing mechanical reperfusion by primary PCI)—is most important in achieving good outcomes for STEMI patients [26, 27, 30, 31]. Another factor, DTB times, is a useful predictor of morbidity and mortality in STEMI patients undergoing primary PCI [32, 33]; a DTB time of 90 min during primary angioplasty is considered a desirable time [26, 34], but shorter times are preferable, and longer times can result in poor clinical outcomes [26, 34]. Experimental studies and human clinical studies have documented that total ischemic time is better correlated with infarct size and mortality than are subinterval times, such as DTB time [31, 35]. Our work revealed that total myocardial ischemia time and DTB time were significantly longer in STEMI patients during the COVID-19 pandemic period than in the non-COVID-19 pandemic period, while the all-cause mortality and MACE were not significantly different between the two periods. Concurrently, the frequency of thrombolytic therapy use in NSTEMI patients during the COVID-19 pandemic period increased in accordance with the modified guidelines for AMI treatment. Among the published guidelines in various regions, some mention that STEMI will preferentially receive primary PCI during the COVID-19 pandemic period [36]. Of course, primary PCI should be the first choice when its rapid implementation can be guaranteed. In special cases, when primary PCI must be delayed, thrombolytic therapy can be an alternative treatment after the risk is fully assessed. Overall, based on previous studies and our work, we recommend reperfusion with thrombolytic therapy for STEMI patients during the COVID-19 pandemic period to expedite treatment, as diagnostic testing of COVID-19 will delay implementation of reperfusion therapy through increased DTB and total ischemic times.

### Limitations

This was a single center experience with limited patient number, and it is a retrospective, chart review study, with all the potential for bias in that format.

## Conclusions

COVID-19 pandemic causes treatment delay in AMI patients and potentially leads to poor clinical outcome in NSTEMI patients. Thrombolytic therapy should be initiated without delay for STEMI when coronary intervention is not readily available; for NSTEMI patients, outcomes of invasive reperfusion were better than medical treatment. All hospitals not only must prepare to treat immediate COVID-19 cases but also provide expeditious and appropriate care for patients with acute coronary artery disease.

## Data Availability

All data generated or analyzed during this study are included in this published article.

## Abbreviations

COVID-19: Coronavirus disease 2019
AMI: Acute myocardial infarction
STEMI: Acute ST-elevation myocardial infarction
STEMI: Non-ST elevation myocardial infarction
FMC: First medical contact
DTB: Door-to-balloon
MACE: Major adverse cardiac event
